# Functional genomic approaches to improve crop plant heat stress tolerance

**DOI:** 10.12688/f1000research.19840.1

**Published:** 2019-10-04

**Authors:** Baljeet Singh, Neha Salaria, Kajal Thakur, Sarvjeet Kukreja, Shristy Gautam, Umesh Goutam

**Affiliations:** 1 Molecular Biology and Genetic Engineering, Lovely Professional University, Phagwara, Punjab, 144411, India; 2 School of Agriculture, Lovely Professional University, Phagwara, Jalandhar, 144411, India

**Keywords:** GWAS, VIGS, T-DNA, CRISPR, Heat stress, Functional genomics

## Abstract

Heat stress as a yield limiting issue has become a major threat for food security as global warming progresses. Being sessile, plants cannot avoid heat stress. They respond to heat stress by activating complex molecular networks, such as signal transduction, metabolite production and expressions of heat stress-associated genes. Some plants have developed an intricate signalling network to respond and adapt it. Heat stress tolerance is a polygenic trait, which is regulated by various genes, transcriptional factors, proteins and hormones. Therefore, to improve heat stress tolerance, a sound knowledge of various mechanisms involved in the response to heat stress is required. The classical breeding methods employed to enhance heat stress tolerance has had limited success. In this era of genomics, next generation sequencing techniques, availability of genome sequences and advanced biotechnological tools open several windows of opportunities to improve heat stress tolerance in crop plants. This review discusses the potential of various functional genomic approaches, such as genome wide association studies, microarray, and suppression subtractive hybridization, in the process of discovering novel genes related to heat stress, and their functional validation using both reverse and forward genetic approaches. This review also discusses how these functionally validated genes can be used to improve heat stress tolerance through plant breeding, transgenics and genome editing approaches.

## Introduction

**Figure 1.**
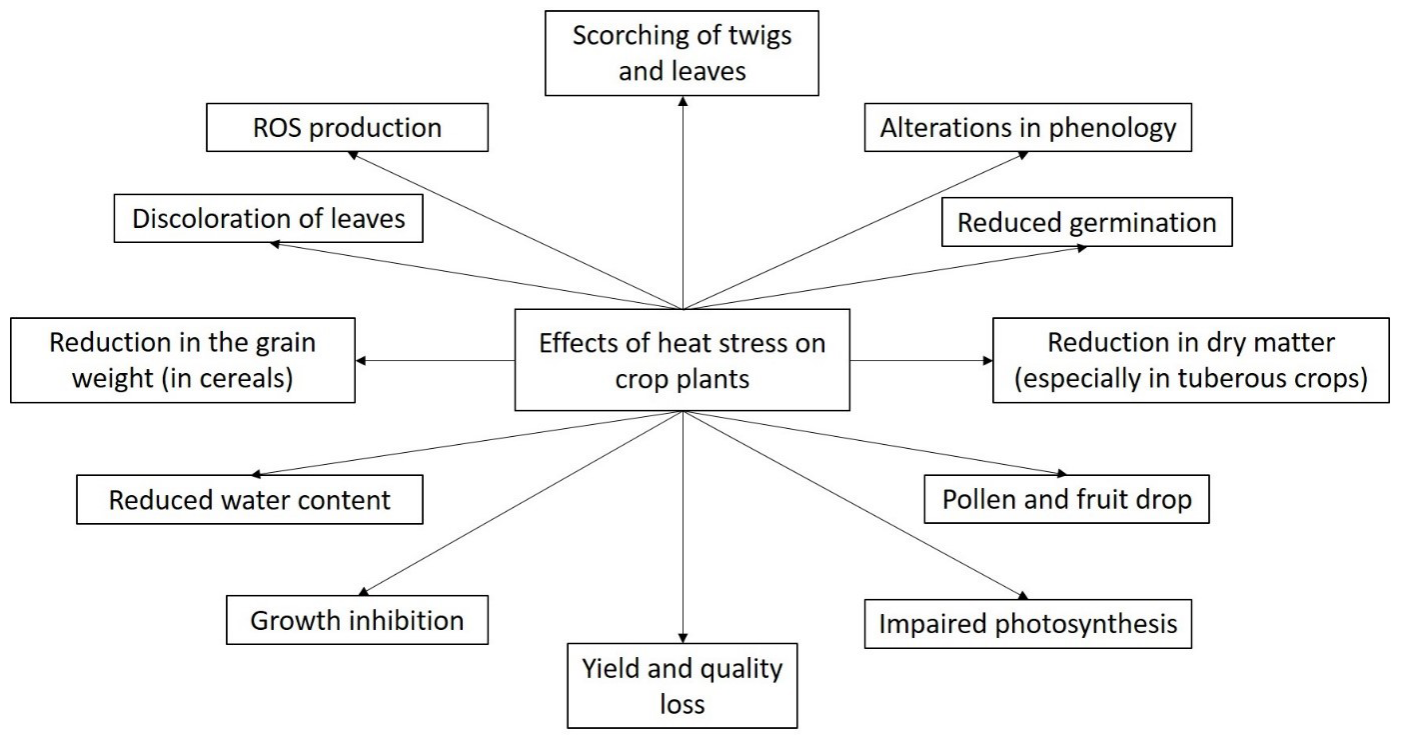
General effects of heat stress on crop plants.

**Figure 2.**
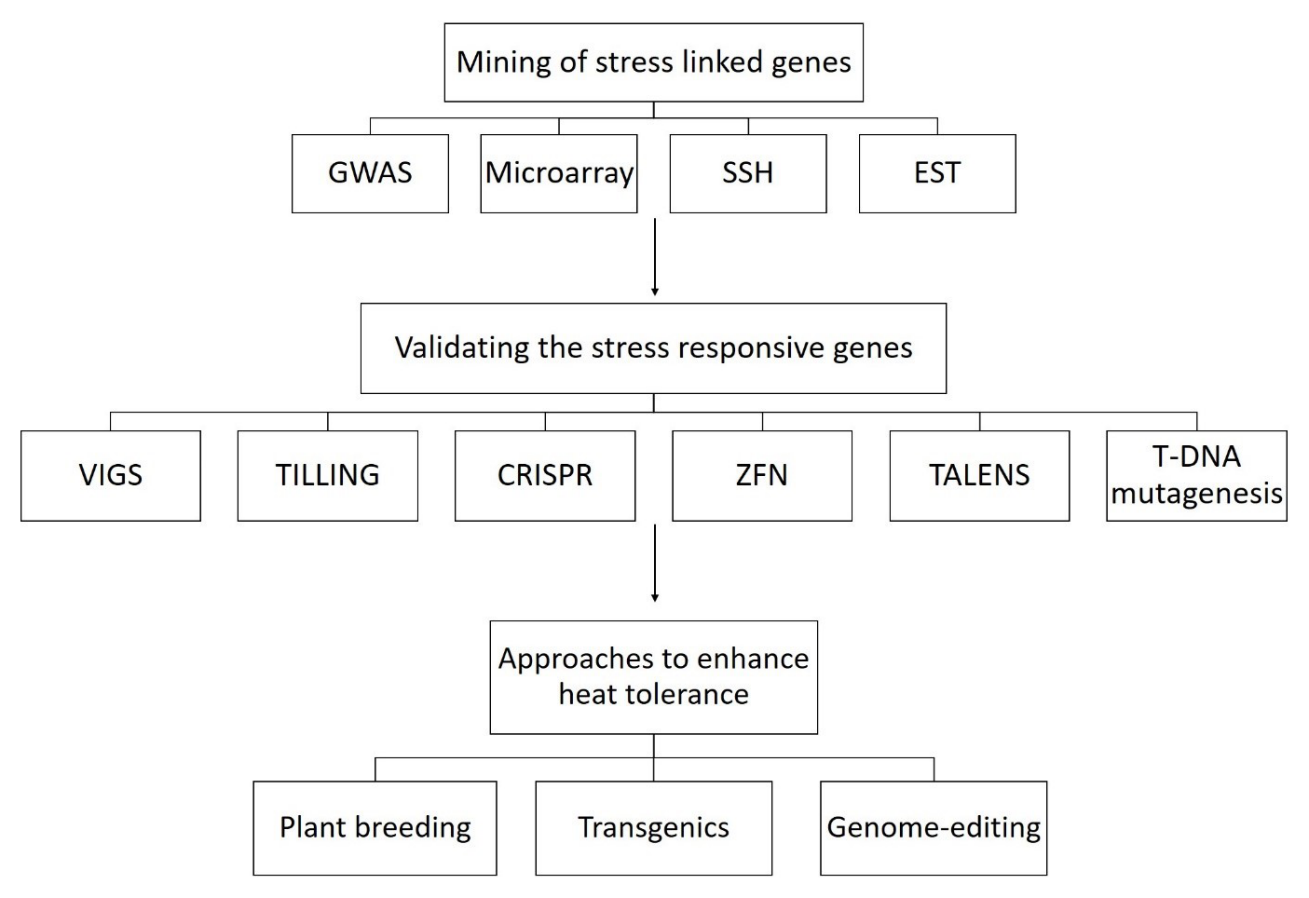
A systematic flow chart depicting the approaches used for the mining of genes associated with heat stress, for the functional validation of candidate genes and approaches that can take advantage of functionally validated genes to increase heat stress tolerance.

Abiotic stresses have numerous adverse effects on crop plants, which further lead to yield and quality losses ( Figure 1). To feed the whole world in the scenario of the changing climate, new and better heat tolerant varieties of various crops is needed ^1^. The understanding of various physiological, molecular and biochemical pathways can facilitate the development of superior heat tolerant varieties ^2^. However, previous efforts, aimed at improving plant heat stress tolerance, have had limited success ^3, 4^ because of the poor understanding of the genetics of heat tolerance. Fortunately, nowadays reference genomes of major food crops and model plant species are available publicly, which provide a solid platform for crop improvement. Moreover, wild species and various landraces of various crops have unknown heat tolerant genes that should be identified and incorporated to high yielding modern cultivars ^5^. The functional genomic approaches such as genome wide association studies (GWAS) and gene expression profiling using microarrays can catalyse the discovery of novel genes associated to heat stress ^6– 8^. In addition, suppression subtractive hybridization (SSH) is another effective and productive technique used for the screening and cloning of the genes/ESTs that express differentially under heat stress ^9, 10^. Reverse genetic techniques can improve the understanding of their expression patterns under heat stress. The plant breeding strategies and new biotechnological tools including genome editing techniques can use these validated genes to enhance heat stress tolerance in crop plants ( Figure 2).

## Mining of stress linked genes

Present crop varieties have limited heat tolerance because earlier domestication, green revolution and conventional breeding were focused to increase yield and qualitative traits ^11^. However, the knowledge of genes/markers/QTL regions associated to heat tolerance is now required to improve thermo tolerance. Previous studies suggested that vast genetic diversity still exists in the germplasms of various crops ^12– 14^. GWAS emerged as a powerful tool to identify the genetic basis behind complex phenotypic traits ^15, 16^, and it provides high mapping resolution compared with conventional genetic mapping ^17, 18^. So far this approach has been applied to major food crops, including wheat ^7, 19, 20^, rice ^21^, maize ^22^, sorghum ^23^and *Brassica napus* L. ^24^, to identify the natural variation associated with heat stress and to understand this genetic basis. Another way to identify and understand the key molecular mechanisms in response to heat stress is a transcriptomic study ^25, 26^; plants respond to heat stress by inducing various heat responsive genes, thus transcriptomic studies provide an effective screening of heat responsive candidate genes ^6^. For example, microarray studies allow the screening of genes on the basis of their expression patterns under stressed conditions at a particular plant developmental stage ^6, 26, 27^. Singh *et al*. (2015) ^6^ investigated the heat responsive genes for potato tuberization and Ginzberg *et al*. ^28^ identified the candidate heat responsive genes for potato periderm formation using microarrays. In addition, SSH is an easy and efficient approach for the identification of genes/ESTs with differential expression under heat stress. This technique is preferred when the genome sequence information is not available ^9^. It can identify the tissue specific differentially expressed transcripts. To identify heat responsive ESTs cDNA libraries can be generated from plants grown under heat stressed conditions ^29^. For example, SSH library of potato skin present 108 candidate genes for suberin and periderm formation ^30^. To investigate the genes/ESTs involved in heat tolerance at the stage of grain filling in wheat, SSH library was constructed by using the leaf RNA samples from heat stressed plants ^9, 29^. The results of these studies provided many heat responsive genes/ESTs, which can be used to develop thermo tolerant wheat varieties.

## Validating the stress responsive genes

The above approaches can identify potential candidate genes linked to heat stress tolerance. However, the functions of the candidate genes must be validated before incorporating them into present cultivars. Both forward and reverse genetic approaches can be employed for functional validation of genes (see examples in Table 1). Forward genetics detect variations in the nucleic acid sequence responsible for a given phenotype ^31^, while reverse genetics detect the gene’s functionality by observing the change in the phenotype due to alterations in known genetic sequence ^32^.

**Table 1.** Some examples of successfully validated potential heat tolerant genes in model plants and major crops.

Plant/crop	Gene	Technique used	Reference
*Arabidopsis thaliana*	*HSF1 and HSF3*	Transcription control (genetic engineering using protein fusion)	33, 34
	*DREB2A CA*	Microarray	35
	*Hsp70*	antisense gene approach	36
	*ATHSF1 (HSF)*	Recombinant DNA technology	33
	*FAD7*	T-DNA	37
	*HSP101*	Transformation	38
Rice *(Oryzasativa)*	*spl7*	Transcription control	39
	*Athsp101*	Agrobacterium mediated transformation	40
Wheat *(Triticumaestivum)*	*TamiR159*	MiR159 (miRNA)	41
	*TaGASR1*	Agrobacterium mediated transformation	42
Carrot *(Daucuscarota)*	*Hsp17.7*	Hsps and molecular Chaperones	43
Chilli pepper *(Capsicum annuum)*	*CabZIP63*	Virus induced gene silencing	44
	*CaWRKY40*	Virus induced gene silencing	45
Tomato *(Solanumlycopersicum)*	*Hsa32*	Subtracted cDNA libraries	46
	*MT-sHSP*	freezing transformation method	47
	*ATG5, ATG7, NBR1, WRKY33*	Virus induced gene silencing	48
	*2-CP1, 2-CP2, 2-CP1/2, ATG5, ATG7*	Virus induced gene silencing	49
	*RBOH1, MPK1, MPK2*	Virus induced gene silencing	50
	*Hsc70.1*	Virus induced gene silencing	51
	*SlLrgB*	RNA interference	51
Barley *(Hordeumvulgare*)	*APX 1*	Agrobacterium mediated transformation	52

Virus-induced gene silencing (VIGS) is a rapid, efficient and cost effective post-transcriptional gene silencing (PTGS) technique used to study target gene(s) functionality ^53^. It can be used as both the forward and reverse genetic approach ^48, 54^. Plants can sense and then respond to heat stress by activating various transcriptional cascades ^55^. Being a PTGS technique, VIGS can be used to knockdown the expression of target genes after transcription. VIGS takes an advantage of a plant’s innate defence mechanism against virus infection. In this technique, a fragment of the target gene is first inserted into a suitable viral vector and then that vector is transformed into the plant, where the viral genome harbouring the fragment of target gene start replicating and produce dsRNA. Then an enzyme DICER cut this dsRNA into multiple siRNA of about 21 nucleotide long. Later these siRNA unwind into two single stranded RNAs, one out of which is degraded and the other one binds to RNA induced silencing complex (RISC), which later degrade the targeted endogenous gene and the effect on gene knockdown can be observed on by phenotypic analysis ^56– 58^. Many candidate genes and transcriptional factors associated with heat stress response/tolerance have been validated successfully through VIGS ^58, 59^. For example, TRV-VIGS based silencing of CabZIP63 gene lowered the tolerance to heat stress in pepper plants, suggesting that CabZIP63 is a positive regulator for thermos tolerance ^44^. The functionality of ATG5, ATG7,FAD7 and NtEDS1genes in response to heat stress have been successfully studied in tomato using tobacco rattle virus (TRV) based VIGS technique ^48, 60^. Recently, VIGS has also been employed to investigate the involvement of small heat shock proteins (CaHSP16.4 and CaHsp25. 9) in heat stress tolerance ^61, 62^.

The role of candidate genes in heat stress tolerance can also be verified through the generation of transfer (T)-DNA mutants ^63^. Like VIGS, insertion of T-DNA in the target gene’s sequence disrupt its functionality, which results in the change of phenotype. This approach is widely accepted because of the genome wide distribution of transposable elements with superior insertions in the gene sequences, resulting in the direct gene knockout ^64^. In addition, T-DNA can also be used as a gain of function approach to study the target gene’s functionality, called as activation tagging ^65^. For example, T-DNA having a tetramer of cauliflower mosaic virus 35S promoter can cause gene activation mutations ^65^. Since plant responses to environmental stresses are polygenic and complex traits, model plants, such as Arabidopsis, are used to first study adaptive responses ^66^. T-DNA mutant lines of many heat tolerant ecotypes of Arabidopsis have been discovered and are available at Nottingham Arabidopsis Stock Centre (NASC) or The Arabidopsis Information Resource (TAIR).

Targeting induced local lesions in genome (TILLING) is another non-transgenic approach that allows the PCR based identification of directed mutations in the target gene sequence and the function of target gene can be analysed from the modified phenotype due to that mutation ^67, 68^. It is a fast and cost efficient technique for the screening of point mutations and for the functional validation gene of interest ^69^. It take advantage of conventional insertional mutagenesis and availability of genomic sequences ^70^. These point mutations can be generated with the help of chemical mutagens such as ethyl methane sulfonate (EMS) ^71^. Nowadays, the genome sequences of many crop plant species are available, which make this technique more effective. The plants under heat stress exhibit different phenotypes associated with allelic variations in their genomic sequence. The TILLING approach used to study these natural variations or SNP mutations in individuals is called as EcoTILLING. The next generation sequencing techniques allow inexpensive TILLING by sequencing method to screen SNP variations ^72^. Recently, the functionality of heat shock binding protein 1 (HSBP1) was examined with TILLING. The chemically induced mutations disrupted the functionality of HSBP1 partially and the mutant plants exhibited increased heat stress tolerance. These findings confirmed that HSBP1 is a negative regulator of heat stress response in tomato ^73^.

In addition, the genome-editing techniques such as transcription activator-like effector nucleases (TALENs), zinc finger nucleases (ZFNs) and clustered regularly interspace short palindromic repeat (CRISPR) can also be used as reverse genetic approaches to study the target gene function. TALENs using sequence specific nucleases (SSNs) became a powerful genome editing technique, which can also be applied as a reverse genetic approach to understand the function of a target gene. It consists of one customizable DNA-binding domain and a nuclease, which generate double stranded DNA breaks (DSBs) at the target gene sequence ^74^. Similar to CRISPR, these DSB are repaired either via NHEJ pathway or via homologous recombination. Both these recovery pathways allow insertion, deletion and intentional replacements in the target gene sequence. These modifications in the target gene’s sequence may cause a variation in the phenotype, which suggests the function of that gene ^74, 75^. The ZFNs are the synthetic proteins having a DNA binding domain that consists two finger modules and a DNA cleaving domain. ZFNs causes DSBs in the targeted DNA sequence and facilitate site-specific mutagenesis, or base substation, which alter or may knockout the gene expression ^76^. The ZFNs have revealed the function of various genes in model plants as well as in crop species ^77^.

Among all genome-editing techniques, CRISPR-Cas9 has emerged as a powerful tool for precise genome editing to study the molecular pathways linked to heat stress and to enhance thermo tolerance in crop plants ^78, 79^. It is comparatively simpler, more accurate and faster than other genome editing techniques. In brief, CRISPR involves designing of a guide RNA of ~20 nucleotides complementary to the gene of interest and a Cas9 nuclease enzyme that cut 3–4 bases next to the protospacer adjacent motif, which is later repaired either by homology directed repair pathway or via error prone non-homologous end joining ^80, 81^. Therefore, this technique can be used to generate gene knockout mutant lines to study the function of targeted gene(s). For example, annexin gene OsAnn3 knockout mutant lines developed via CRISPR-Cas9 technique revealed the role of OsAnn3 gene in cold stress tolerance in rice ^82^.

## Approaches to enhance heat tolerance

Plants have inherent mechanisms to survive under heat stressed conditions but the heat tolerance capacity of plants varies species-to-species and even within the species. If heat tolerant genes are present in sexually compatible species, then marker-assisted selection (MAS), new generation molecular breeding, precision breeding and genome editing techniques can be used.

Thermo tolerance is a complex multigenic trait, which is influenced by genotype X environment interactions ^83^. Development of heat tolerant crop varieties through traditional breeding is very labours and time consuming. However, precision breeding with the help of MAS can accelerate the plant breeding programs with high efficiency ^84^. SNPs and simple sequence repeats (SSR) are being used widely in plant breeding experiments aimed to enhance abiotic stress tolerance. Presently the use of SNPs become more common in plant breeding than SSR markers ^85, 86^. Garg *et al*. ^87^ found one SNP in the sequence of heat shock protein (HSP16.9) between a heat tolerant and heat susceptible wheat genotypes. This SNP contribute 29.89% phenotypic variation for grain weight per spike. Recently, many SNPs associated to heat stress tolerance have been identified in major food crops ^7, 88– 90^. However, heat stress tolerance is a polygenic trait and a single molecular marker contribute little to improve it. Therefore, it is important to incorporate several SNPs associated to various QTLs that are controlling the heat stress tolerance mechanisms ^89, 91^. However, the accessibility of genome editing techniques opened various new windows to introduce targeted editing of plant genomes to understand the molecular aspects involved in heat stress tolerance ^92, 93^. For example, ethylene response factors (ERFs) are the stress induced transcriptional factors that take part in abiotic stress tolerance. The CRISPR-Cas9 based genome editing of one such ethylene response factor from AP2/ERF superfamily enhanced abiotic stress tolerance in crop plants ^94^.

In cases when heat tolerant genes do not exist in sexually compatible species, these methods cannot be applied. Advanced biotechnological tools can increase the limited heat stress tolerance in crop plants. The transfer of heat tolerant genes through recombinant DNA technology can generate heat tolerant transgenic lines in a short amount of time. This method also allows utilisation of potential genes from other species to enhance thermo tolerance in target crops, e.g. *AmDREB2C*, from *Ammopiptanthus mongolicus* has been used to increase heat stress tolerance in transgenic *Arabidopsis* plants ^95^. In addition, many genes responsible for heat stress tolerance have been identified and validated in model plants and also in major food crops that can be introduced to heat susceptible cultivars or their expression levels can be increased by generating their stable overexpression lines. For instance, the overexpression TaPEPKR2 gene enhanced heat stress tolerance in wheat and Arabidopsis plants ^96^.

## Conclusion

Heat stress affects crop production significantly. Plants respond to heat stress by activating complex molecular networks, such as signal transduction, metabolite production and expressions of heat stress-associated genes. With the developments in plant functional genomics techniques, many novel genes related to heat stress tolerance have been identified and are being used to improve stress tolerance with the help of advanced biotechnological approaches. Next generation sequencing and genome-editing techniques will play crucial roles in crop improvement. In the near future, the scientists will have a better understanding of plant heat tolerant mechanisms and farmers will be able to grow better high yielding heat tolerant crop varieties in the fields.

## Data availability

No data is associated with this article.
