# Supplementary material for: Functional genomic approaches to improve crop plant heat stress tolerance
Source: F1000Res. 2019 Oct 4;8:1721. [Version 1] doi: 10.12688/f1000research.19840.1 (PMC6896246; doi:10.12688/f1000research.19840.1)
Supplement: Peer review history

# Peer review: Reviewer response for version 1

**DOI:** 10.5256/f1000research.21768.r56662

**Published:** 2020-01-09

**Authors:** Ajaya Biswal

**Affiliations:** 1 Department of Biochemistry and Molecular Biology, University of Georgia, Athens, GA, USA

Plants are constantly exposed to different abiotic stresses. They have developed a wide variety of adaptation mechanisms through molecular, physiological, and biochemical alterations to cope with different stress conditions. Heat stress is still considered as one of the major limiting factors for crop yields. Simultaneously, heat stress significantly contributes to climate change. This review well describes the wide potential of various functional genomics strategies from gene selection and identification of heat tolerance genes to genetic manipulation by genetic engineering and genome editing.  However, I have a few comments that need to be addressed:

1. The authors should have discussed the functional role of heat shock protein (HSPs) and molecular chaperones under extreme temperatures. I believe a potential discussion about the possible role of HSP/chaperones towards heat stress adaptation to plants will strengthen this review.
2. I do not find a specific reason for selecting tolerant genes from a few selected crops/plants (Table I). I encourage to refer a number of heat-tolerant gene lists from other major crops.

I confirm that I have read this submission and believe that I have an appropriate level of expertise to confirm that it is of an acceptable scientific standard, however I have significant reservations, as outlined above.

# Peer review: Reviewer response for version 1

**DOI:** 10.5256/f1000research.21768.r56551

**Published:** 2019-12-05

**Authors:** Neila Abdi

**Affiliations:** 1 Field Crop Laboratory, National Institute of Agricultural Research of Tunisia (INRAT), Tunis, Tunisia

During review process, I appreciated this study. Authors discuss new approach that is well written. However, I found following points to be check for further improvement in manuscript:

- Abstract: Please correct "in this area" instead of "in this era".
- Introduction: Please replace "tolerant varieties" instead of "new and better heat tolerant varieties".
-  The conclusion need to be ameliorate because it is very brief.

The review discussed very well and comprehensively the context of  current literature.

 All statements are correct and author can refer to others citations to make the paper more supported.

 The review is written with good English and it did not need any improvement.

 Author can refer the following paper to explain more:

- Zafar S.A., Zaidi S.S.A., Gaba Y., Singla-Pareek S.L., Dhankher O.P., Li X.,  Mansoor S., Pareek A (2019). Engineering abiotic stress tolerance via CRISPR/ Cas-mediated genome editing. Journal of Experimental Botany, doi:10.1093/jxb/erz476, pp 1-10 ^1^.
- Cushman J.C., Bohnert H.J. 2000. Genomic approaches to plant stress tolerance. Genome studies and molecular genetics, 3:117–124 ^2^.

In general, this review can be accepted with minor revision.

I confirm that I have read this submission and believe that I have an appropriate level of expertise to confirm that it is of an acceptable scientific standard.

## References

[1] : Engineering Abiotic Stress Tolerance via CRISPR-Cas mediated genome editing. *J Exp Bot*.2019; 10.1093/jxb/erz476 10.1093/jxb/erz476 31644801

[2] : Genomic approaches to plant stress tolerance. *Curr Opin Plant Biol*.2000;3(2) : 10.1016/s1369-5266(99)00052-7 117-24 10.1016/s1369-5266(99)00052-7 10712956

# Peer review: Reviewer response for version 1

**DOI:** 10.5256/f1000research.21768.r54724

**Published:** 2019-10-28

**Authors:** Himanshu Sharma

**Affiliations:** 1 Agri-Biotechnology Department, National Agri-Food Biotechnology Institute (NABI), Mohali, Punjab, India

The review entitled “Functional genomic approaches to improve crop plant heat stress tolerance” is relevant considering the present climate change scenario and global food security. The review highlighted the negative effects of heat stress on crop plants. Heat stress is a major yield-limiting factor in this climate change scenario. This review presents recent achievements in understanding the molecular basis of heat tolerance in crop plants. Further, it discussed the usage of appropriate approaches from the identification of heat tolerant genes to their incorporation into cultivated crop varieties. The article is written and organised nicely but still some minor improvements can be done.

**Specific comments**

Title

 The title of the article is appropriate and accurately reflect the content of the paper.

Abstract

 It represent the article nicely.

Introduction

 Last two lines of introduction can be improved. Authors written reverse genetic techniques can improve the understanding of their expression patterns under heat stress. I suggest that instead of

 writing reverse genetic techniques they should mention the names of these techniques.

Mining of stress linked genes

 Authors mentioned: Singh et al. (2015) investigated the heat responsive genes for potato tuberization and Ginzberg et al. identified the candidate heat responsive genes for potato periderm formation using microarrays.

 I suggest, either write the year with Ginzberg et al. or remove year from Singh et al. (2015) to keep the same formatting.

Validating the stress responsive genes

 I found this section is very interesting.

Approaches to enhance heat tolerance

 Do not write the gene name; AmDREB2C in Italics as rest of the gene names in text are not written in Italics.

Conclusion

 Summarized the article in a coherent way.

 Overall, the article is logically structured and well-organised, and provides a useful compilation of subject matter related to addressed topic. It is an important contribution and I highly recommend it.

I confirm that I have read this submission and believe that I have an appropriate level of expertise to confirm that it is of an acceptable scientific standard.
